# Eating Disorders and Diabetes: Facing the Dual Challenge

**DOI:** 10.3390/nu15183955

**Published:** 2023-09-12

**Authors:** Magdalena Dziewa, Bartosz Bańka, Mariola Herbet, Iwona Piątkowska-Chmiel

**Affiliations:** Chair and Department of Toxicology, Faculty of Pharmacy, Medical University of Lublin, Jaczewskiego 8b Street, 20-090 Lublin, Poland

**Keywords:** eating disorders, diabetes, mental disorders, integrated care, healthcare challenges

## Abstract

Eating disorders and diabetes mellitus are distinct yet closely linked health conditions, presenting distinct challenges in terms of care and management. Eating disorders encompass a spectrum of mental health disorders characterized by abnormal eating behaviors and disruptions in weight regulation. Research indicates that individuals with diabetes might be at an elevated risk of developing eating disorders. The necessity to adhere to specific dietary guidelines, monitor blood sugar levels vigilantly, and manage drug administration can collectively contribute to the emergence of detrimental attitudes toward food and body image. On the other hand, incorrect eating behaviors such as binge eating and purging can disrupt blood sugar control, significantly impacting the development and management of diabetes. This intricate relationship emphasizes the crucial necessity for a comprehensive understanding and specialized care to effectively address the dual challenges faced by individuals dealing with both diabetes and eating disorders. This paper represents the inaugural comprehensive review delving into the intricate connection between eating disorders and diabetes, thereby illuminating previously under-researched areas. The insights gleaned from this review may contribute to developing integrated interventions that aim to improve the overall well-being and quality of life for individuals grappling with the complexities of eating disorders and diabetes.

## 1. Introduction

Eating disorders (EDs) are serious and complex mental health conditions that impact an individual’s relationship with food and their body image. Despite the first documented description of an eating disorder over three hundred years ago, the issue continues to persist and even increase globally [1]. Eating disorders affect at least 9% of the global population, making them one of the most prevalent mental health disorders [2,3]. The onset of most eating disorders is typically in adolescence or early adulthood, with a median age of onset ranging from 18–22 [4]. The ratio of males to females in eating disorders ranges from 1:2 to 1:4, depending on the specific diagnosis [5]. The factor determining the higher percentage of morbidity among women is that women set themselves the goal of achieving a slim, slender figure, while men are dominated by the idea of a large, muscular body. Women generally have higher standards and pressure to be attractive. While men have a tendency to consume more foods with high calorific value, women usually restrict their calories [6]. Furthermore, research indicates a higher incidence of eating disorders in women compared to men, which could potentially be attributed to variations in brain function, which might foster the impulsive and repetitive behaviors commonly associated with eating disorders [7,8]. Furthermore, eating disorders exhibit the highest mortality rate among all psychiatric disorders, surpassing even dysthymia, bipolar affective disorder, and schizophrenia, with a rate of 5.6% per decade [9]. A crucial factor contributing to this elevated risk is the significant disparity between men and women when it comes to seeking treatment (American Addiction Centers, n.d.). Specifically, men are notably less inclined to seek help for their eating disorders, which can lead to delayed or inadequate intervention and worsen their overall prognosis.

It is important to note that anorexia nervosa is becoming increasingly prevalent and may soon become the third most common chronic disease in adolescent girls aged 15–19, after obesity and asthma [10]. Statistics reveal that around 26% of individuals grappling with eating disorders make suicide attempts [9,11]. This distressing finding underscores the severity of the mental health challenges faced by those affected by eating disorders and emphasizes the urgent need for effective intervention and support to prevent such tragic outcomes. Shockingly, research shows that there is no shortage of diagnosed individuals as young as nine or ten years old [12]. In the UK, the disorder is even recognized among preschoolers [13]. According to Wojtyła et al. 2011 study [14], approximately 33% of teenagers in Poland have attempted to lose weight using different methods, including drastic measures, and every second girl and every fourth boy believe that they are too fat [15]. Over the last twenty-seven years, Poland has moved up six units in the rankings, placing 106th in the world [16]. Such a significant increase may be due to an increasingly irregular lifestyle associated with excess work and stress, resulting in unsuitable nutritional patterns [17].

The causes of eating disorders are complex and multifaceted and may involve a combination of genetic and environmental psychological factors and vary from person to person. Some individuals may be more predisposed to developing an eating disorder due to genetics, while others may turn to disordered eating as a way to cope with stress, trauma, or other emotional challenges. In recent years, there has been growing interest in epigenetic factors, which may play an important role in both eating disorders and diabetes. These epigenetic modifications may affect the expression of genes that regulate insulin production, insulin sensitivity, appetite regulation, and areas of the brain responsible for reward and emotion [17]. Changes in these genes may contribute to the development of obesity and type 2 diabetes. Growing evidence suggests that epigenetic changes that occur during fetal development in response to factors such as maternal nutrition and stress may increase the risk of diabetes later in life [17,18,19]. Additionally, epigenetic changes may affect the body’s response to stress, which is a known cause of some eating disorders. In addition to genetic and epigenetic influences, psychological factors are crucial contributors to eating disorders [8]. Societal pressure to conform to a certain body type or appearance can also contribute to the development of eating disorders, especially in a world where social media and unrealistic body standards play a significant role in the self-esteem of young people. Additionally, psychological trauma resulting from marginalization by peers, comments about appearance, or comparing body shapes and sizes may also contribute to dissatisfaction with one’s appearance and the development of eating disorders [18].

Eating disorders impose substantial physical and psychological consequences on those affected. Among the common physical repercussions are malnutrition, electrolyte imbalances, and cardiac issues, which can pose serious health risks [19]. In addition to the physical toll, individuals with eating disorders often battle mental health challenges, such as depression and anxiety, further complicating their well-being. Moreover, these disorders can lead to social isolation, as individuals may withdraw from social situations involving food due to their struggles and fears surrounding eating [19]. This social avoidance can exacerbate feelings of loneliness and detachment from others. As a consequence of the combined physical, psychological, and social impacts, those with eating disorders may experience a significant decline in their overall quality of life. The disorders can create difficulties in various aspects of daily living, including relationships, work or school performance, and overall emotional well-being [19].

Recent epidemiological studies have shed light on the heightened risk of developing eating disorders, including bulimia, anorexia, and binge eating disorder, among individuals with diabetes [20,21,22]. It turns out that eating disorders and diabetes mellitus are distinct yet intricately interconnected health conditions. The need to follow precise dietary guidelines, diligently monitor blood sugar levels, and manage medication administration can collectively contribute to the development of detrimental attitudes toward food and body image. Conversely, maladaptive eating behaviors such as binge eating and purging can disrupt blood sugar control, thereby significantly affecting the onset and management of diabetes.

Diabetes and eating disorders create a complex and potentially dangerous combination of conditions that can lead to increased morbidity and mortality. Those with both conditions face the challenge of maintaining a healthy weight and controlling blood sugar levels, creating a vicious cycle that can result in further complications such as cardiovascular disease, nerve damage, and kidney failure [23,24,25]. The psychological toll of eating disorders, such as stress, anxiety, and depression, can further exacerbate diabetes management [24].

The primary objective of this review was to assess the relationship between eating disorders and diabetes mellitus, acknowledging the unique but interconnected nature of these two conditions. The primary objective of this review was to conduct a thorough and comprehensive analysis of the potential relationship between eating disorders and diabetes. Additionally, our objective was to elucidate the factors contributing to the emergence of eating disorders among individuals affected by diabetes. Moreover, we aimed to assess how the control of blood sugar levels and the psychological facets of disordered eating impact the holistic treatment and well-being of those confronting both diabetes and eating disorders. The insights gathered in this review have the potential to offer valuable guidance in shaping comprehensive interventions, which could be of great importance due to their practical implications in clinical medicine. These interventions aim to holistically enhance the overall will lead to improvements in the quality of life and well-being for individuals who grapple with the intricate challenges posed by a dual diagnosis.

## 2. Diabetes and Disordered Eating Behaviors/Eating Disorders

Diabetes, as a chronic metabolic condition, presents unique challenges in relation to the development and management of eating disorders. Over the years, extensive research has provided valuable insights into the prevalence of eating disorders among individuals with diabetes, uncovering significant patterns and risk factors associated with this population [26,27,28].

### 2.1. Disordered Eating vs. Eating Disorder

**Figure 1:**
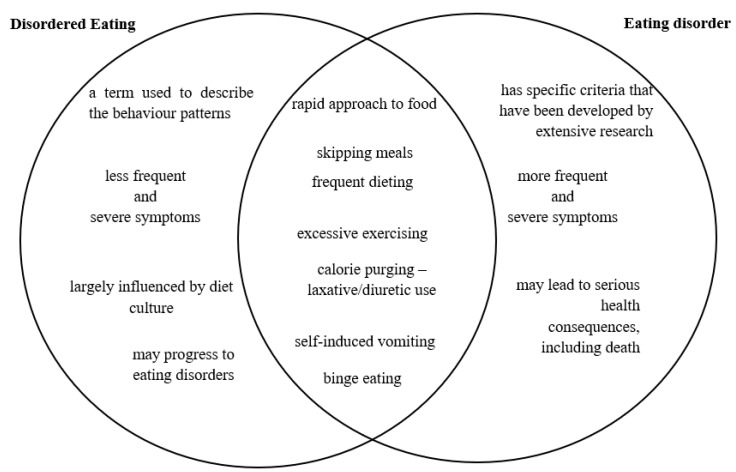
Similarities and differences between disordered eating and eating disorder.

While these terms share similarities, they are not interchangeable, and recognizing their differences is crucial (Figure 1). An eating disorder is considered a clinical diagnosis that involves specific criteria related to the severity, degree, and frequency of disordered eating behaviors. On the other hand, disordered eating encompasses a range of problematic eating behaviors and attitudes that may not yet meet the criteria for a formal eating disorder diagnosis but still have the potential to negatively impact an individual’s physical and psychological well-being.

By understanding the nuances between disordered eating and eating disorders, healthcare professionals can better assess and address the specific needs of individuals with diabetes who may be at different points along the spectrum of these conditions. Early recognition of disordered eating patterns can facilitate timely intervention and support, potentially preventing the escalation to a full-blown eating disorder and improving overall treatment outcomes for those affected by diabetes and these eating-related challenges.

The constant focus on food as a crucial aspect of diabetes management closely ties individuals with diabetes to both disordered eating and eating disorders. Current scientific reports highlight a widespread occurrence of nutrition-related disorders and errors in people with type 1 diabetes, particularly when compared to age-matched individuals without hyperglycemia [29]. The main mistakes in the control and supervision of eating habits and, consequently, eating disorders are inconsistent and irregular insulin treatment. Ultimately, as a result of long-term incorrect treatment, this leads to a lack of glycemic control and, ultimately, an increase in the risk of complications [29]. In individuals diagnosed with both diabetes and an eating disorder, the onset of diabetes typically precedes the development of the eating disorder, especially in cases of type 1 diabetes. However, the reverse relationship may occur with type 2 diabetes, where the presence of an eating disorder, such as binge eating, can potentially contribute to the development of the disease. Among patients with type 1 diabetes, bulimia nervosa is the most common eating disorder observed. This disorder involves cycles of binge eating followed by maladaptive weight control behaviors. In contrast, individuals with type 2 diabetes more frequently exhibit binge eating without purging behaviors and may also experience the syndrome of night eating, characterized by waking up in the middle of the night to consume food. Anorexia nervosa, characterized by severe food restriction, is the least common eating disorder observed in this population but carries an alarmingly high mortality rate.

These are some common signs of disordered eating behaviors that may be observed in individuals with diabetes (Table 1) [30].

Early recognition and intervention are vital for addressing these issues and promoting the well-being of individuals with both diabetes and disordered eating, as these conditions can have significant impacts on their overall health and quality of life. Individuals with diabetes may be at an increased risk of developing eating disorders due to the complex relationship between food, body image, and diabetes management.

One type of eating disorder that may occur in individuals with diabetes involves severe dietary restriction (Table 2). This behavior manifests as severely limiting calorie intake or eliminating essential macronutrients from the diet. The motive behind such restriction is often driven by a desire to control blood sugar levels or achieve weight loss [28]. Objective binge eating is another type of eating disorder observed in individuals with diabetes. It involves consuming an unusually large amount of food within a short period while experiencing feelings of being out of control. The binge episodes may be triggered by various factors and can lead to significant emotional distress. Subjective binge eating refers to losing control over eating without necessarily consuming large quantities of food. Individuals experiencing subjective binge eating may feel overwhelmed by their eating habits and struggle with emotional and psychological consequences, even if the physical volume of food consumed is not as extreme.

In some cases, individuals with diabetes and an eating disorder may resort to other maladaptive weight control strategies. This can include restricting or manipulating life-saving insulin doses in an attempt to achieve weight loss. Such behaviors can be dangerous and have serious health implications.

### 2.2. Eating Disorders in Type 1 Diabetes

A comprehensive investigation conducted by Jones et al. 2000 [31] shed light on the prevalence of eating disorders among females with type 1 diabetes, revealing that only 10% of them were affected. These findings indicate that women diagnosed with type 1 diabetes are at a 2.4-fold higher risk of developing eating disorders compared to those without diabetes. Grylli et al. 2004 [32] found that 11.5% of female participants with type 1 diabetes had eating disorders, while none of the male participants exhibited such conditions. Similarly, an analysis of studies focusing on disordered eating behavior in adolescents and young adults showed a higher prevalence of such behaviors among individuals with type 1 diabetes compared to their peers [31,33].

However, Meltzer et al. [34] presented contrasting results in their analysis. They suggested that individuals with diabetes, especially females, do not exhibit a higher susceptibility to eating disorders. In fact, females with diabetes reported lower levels of body dissatisfaction compared to the general population. Nonetheless, authors highlighted a heightened risk of developing disordered eating patterns among female patients aged 13–14 years [34].

Based on the available evidence, there is a suggestion that Type 1 Diabetes is more frequently observed prior to the development of restrictive eating disorders, particularly Anorexia Nervosa (AN) or Avoidant Restrictive Food Intake Disorder (ARFID) [31]. This finding indicates that individuals with Type 1 Diabetes may be at a higher risk of subsequently developing these specific forms of eating disorders.

#### 2.2.1. Anorexia Nervosa

Anorexia nervosa (AN) is a serious eating disorder with significant physical and psychological consequences. It is characterized by extreme food restriction, a distorted body image, and a deep fear of gaining weight (Table 3). Individuals with AN often have low body weight and severe malnutrition, and they may engage in excessive exercise or harmful purging behaviors, such as self-induced vomiting and laxative abuse [35].

Several risk factors contribute to the development of AN. Those with a family history of AN or comorbid mental health conditions such as depression, anxiety, and substance abuse are at a higher risk of developing the disorder. Research indicates that individuals with a first-degree relative who has AN have a 10-fold greater risk of developing the illness themselves [36,37,38]. Additionally, social and environmental factors, such as societal pressure to conform to a specific body type, lack of support systems, and traumatic events, can also play a role in the onset of AN [35].

Adolescent girls and young women affected by AN often experience numerous physical complications. These may include skin issues such as xerosis and acne, telogen effluvium (hair loss), gastric problems such as dilatation with evidence of mucosal necrosis, arrhythmias (irregular heartbeats), acrocyanosis (bluish discoloration of extremities), sodium depletion, hypovolemia (low blood volume), and associated hemoconcentration (increased concentration of blood cells and plasma). Moreover, AN can lead to bone demineralization due to estrogen deficiency and body weight loss, which are likely the main factors contributing to the development of osteoporosis and osteopenia [39].

Incidence rates of anorexia nervosa (AN) can vary based on the study population and healthcare setting [40]. However, there are reports indicating an increase in AN cases among adolescent girls aged 10–14 years [40,41]. A primary healthcare research study in Great Britain involving individuals aged 13–16 revealed a rise in diagnosed cases of AN in 2018 compared to analyses conducted in 2003 [16]. Current data suggest that up to 4% of females and up to 0.3% of males may experience AN at some point during their lives [16]. It is important to note that AN has the highest mortality rate of all psychiatric disorders and is associated with increased suicidality [13]. Early recognition and treatment of AN are crucial to prevent severe physical and psychological consequences.

**Figure 2:**
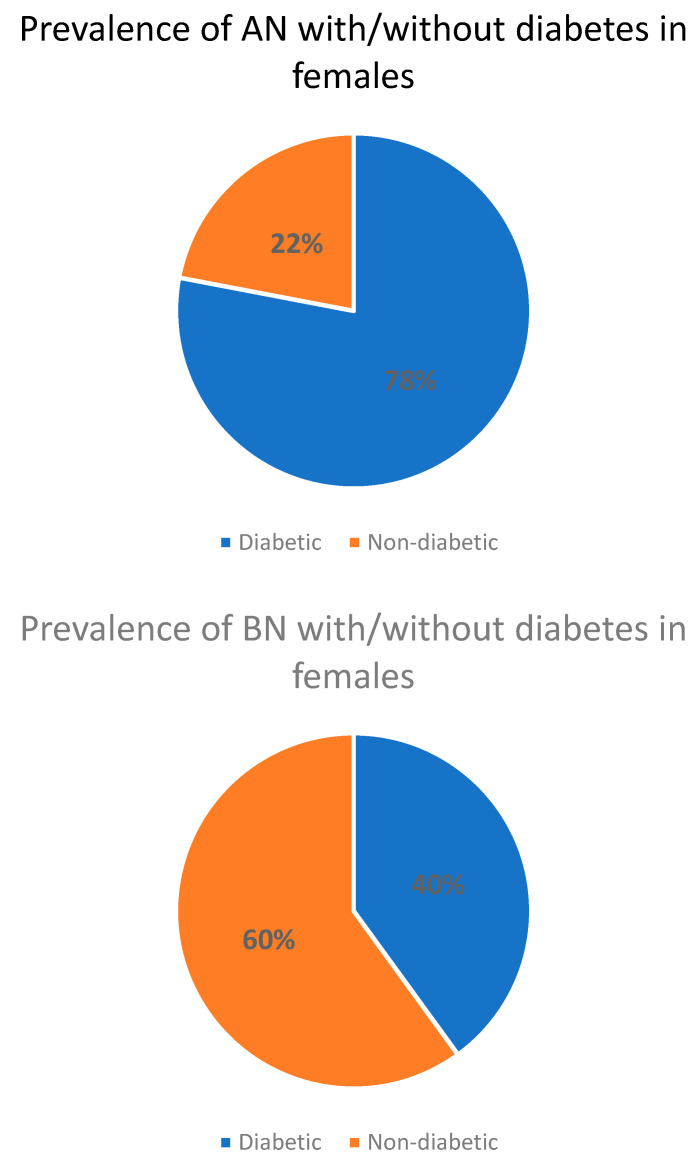
Prevalence of AN or BN with/without diabetes [42].

Both diabetes mellitus and AN share a heightened awareness of carbohydrate consumption. When type 1 diabetes and AN are combined, publicly available data indicate elevated HbA1c levels and an increased risk of various acute and chronic complications [24]. The lack of stability and regularity in the patient’s eating pattern is a common link between both disorders. Some studies have shown an increased risk of eating disorders, including AN, in individuals with diabetes, but the prevalence rates of comorbid AN with diabetes may not necessarily be significantly higher than in patients without diabetes (Figure 2) [42].

Managing the coexistence of diabetes and AN necessitates a comprehensive approach that addresses the unique challenges posed by both conditions. Early identification, intervention, and ongoing support are crucial to improving the outcomes and well-being of individuals facing this complex dual diagnosis.

#### 2.2.2. Avoidant/Restrictive Food Intake Disorder

Avoidant/Restrictive Food Intake Disorder (ARFID) is a distinct eating disorder characterized by limited interest or a narrow range of food choices, often driven by sensory issues or negative experiences [43]. Unlike picky eating in preschoolers, restrictive eating behaviors in ARFID are not motivated by body image disturbances or a desire to lose weight (Table 2). Individuals with ARFID may experience severe malnutrition and disruptions in psychosocial functioning as potential consequences.

The prevalence rates of ARFID vary across different populations. In clinical eating disorder populations, the rates range from 1.5% to 64%, as reported by several studies [44,45,46,47,48,49,50,51]. However, in non-clinical populations, the rates are generally below 15.5% [52,53,54]. One noteworthy aspect is that ARFID primarily affects younger patients, and surprisingly, in comparison to other eating disorders, there is a significantly higher proportion of males affected by ARFID [55]. This highlights the importance of recognizing the unique features and demographics of ARFID to provide appropriate and targeted interventions for those affected.

#### 2.2.3. Bulimia Nervosa

Bulimia Nervosa (BN) is a serious eating disorder characterized by a cycle of binge eating followed by purging behaviors. Binge eating episodes involve consuming large amounts of food within a short period, and purging behaviors can include self-induced vomiting, the use of laxatives, enemas, or excessive exercise [56]. Individuals with bulimia nervosa may have a normal or slightly above-average body weight (Table 3).

The etiology of bulimia nervosa is multifaceted, involving a combination of factors such as personality traits, biology, sociocultural influences, behavioral patterns, and family dynamics [56,57]. There are two types of bulimia: laxative and non-laxative. Laxative bulimia involves the use of drugs and enemas in addition to self-induced vomiting, while non-laxative bulimia involves regular excessive exercise and restrictive eating.

If left untreated, bulimia nervosa can have significant negative consequences on both mental and physical health. Physical complications may include weakness, diarrhea, constipation, headaches, anemia, dental issues such as the destruction of tooth enamel, gastrointestinal problems such as gastroesophageal reflux disease, esophagitis, and gastritis, as well as nutritional deficiencies [56].

Bulimia nervosa can affect individuals of all ages and genders, but it is most prevalent in the 15–29-year-old age group. The incidence of bulimia nervosa is generally lower in men than in women, with a sex ratio of about 1:10 [58,59]. Studies have shown that up to 3% of females and more than 1% of males may be affected by bulimia nervosa [60].

The mortality risk associated with bulimia nervosa is comparable to that of anorexia nervosa and is linked to cardiovascular problems [61], a higher risk of suicide [11,62], as well as other complications involving the gastrointestinal, endocrine, and renal systems [63].

#### 2.2.4. Diabulimia

Diabulimia is a dangerous and concerning eating disorder commonly observed in individuals with type 1 diabetes. It involves the intentional omission or reduction in insulin to lose weight or prevent weight gain. As insulin is essential for regulating blood sugar levels in individuals with type 1 diabetes, reducing or omitting insulin leads to elevated blood sugar levels, causing the body to break down fat for energy, resulting in weight loss, which may be the desired outcome for some individuals. Several studies have reported that the fear of weight gain is a core component in the emergence of insulin mismanagement [64,65]. However, the act of reducing or omitting insulin can have severe consequences. Prolonged high blood sugar levels can lead to diabetic ketoacidosis, a life-threatening condition requiring immediate medical attention. Additionally, persistently elevated blood sugar levels can lead to various complications, such as nerve damage, kidney damage, and vision problems [28]. The symptoms of diabulimia can vary depending on the duration and severity of the condition (Table 4).

Diabulimia is often linked to emotional and psychological difficulties [67]. Individuals with diabetes who engage in diabulimic behaviors may experience intense guilt, shame, and anxiety concerning their eating habits and diabetes management. The complex interplay between diabetes and disordered eating can create a detrimental cycle where the pursuit of weight control leads to worsened diabetes management, an increased risk of complications, and further deterioration of psychological well-being.

The consequences of diabulimia are severe, and individuals who omit insulin are found to be at three times greater risk of premature death than those who adhere to their prescribed treatment. Early detection and comprehensive intervention are crucial to addressing diabulimia and providing individuals with the necessary support to manage both their diabetes and disordered eating behaviors.

### 2.3. Eating Disorders in Type 2 Diabetes

Individuals with type 2 diabetes are also susceptible to the risk of developing eating disorders. Patients with type 2 diabetes often experience overweight or obesity, which may be correlated with bad eating habits and episodes of binge eating. These behaviors can negatively impact blood sugar control and overall health [68].

Some individuals with type 2 diabetes may have irregular meal timing, such as skipping meals or eating at inconsistent intervals. Irregular meal timing can lead to unstable blood sugar levels, making it challenging to achieve glycemic control. Skipping meals, especially breakfast, can affect insulin sensitivity and increase the risk of hypoglycemia or hyperglycemia [28]. Restrictive eating behaviors, excessive exercise, and purging can lead to irregular and unpredictable blood glucose levels. This makes it challenging to maintain stable glycemic control and can increase the risk of hyperglycemia or hypoglycemia. Further, excessive exercise and purging can lead to irregular and unpredictable blood glucose levels. This makes it challenging to maintain stable glycemic control and can increase the risk of hyperglycemia or hypoglycemia. What is more, depressive symptoms and behaviors can contribute to eating disorder development, especially in patients with diabetes (occurrence is two to three times higher—Bădescu et al., 2016) [69]. Individuals with depression experience varying appetite changes due to their depressive symptoms, which may leave them vulnerable to disordered relationships with food.

#### 2.3.1. Binge Eating Disorder

A binge eating disorder (BED) is a significant mental health condition characterized by recurrent episodes of binge eating, where individuals consume large amounts of food in a short period while feeling a lack of control over their eating behavior. Unlike bulimia nervosa, individuals with BED do not engage in purging behaviors such as vomiting or excessive exercise. The WHO Global Mental Health Study found that BED has a higher prevalence rate compared to bulimia nervosa and anorexia nervosa (Figure 1) [70]. Giel et al., 2022 [71] estimated that binge eating disorder (BED) affected around 0.6–1.8% of adult women and 0.3–0.7% of adult men globally between 2018 and 2020. Unlike bulimia, people with binge eating disorders do not engage in purging behaviors [72]. Due to the caloric overconsumption involved, BED is strongly associated with overweight and obesity, which leads to a high body mass index (BMI). Individuals with BED have significantly higher current BMI than respondents without a history of eating disorders: 2.6% (BMI = 30–35) and 4.5% (BMI ≥ 35) in obese subjects, respectively. BED can also lead to medical complications such as type 2 diabetes, high blood pressure, and heart disease [71].

**Figure 3:**
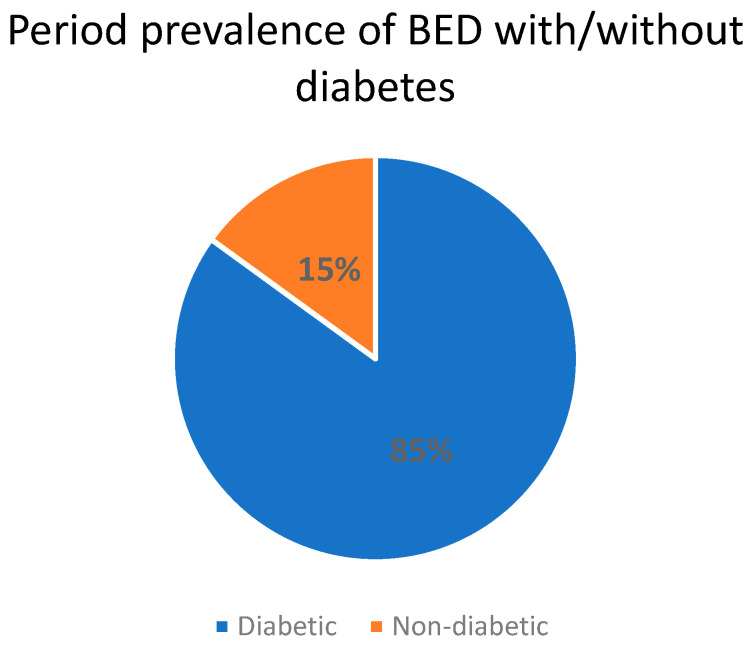
Period prevalence of BED with/without diabetes [77].

Research indicates that binge eating behaviors are observed in approximately 7% to 20% of individuals diagnosed with type 2 diabetes [73,74,75], with around 8% receiving a clinical diagnosis of BED [76]. Furthermore, approximately one-third of individuals with BED also have coexisting type 2 diabetes [77] (Figure 3). Remarkably, a significant proportion ranging from 10% to 40% of individuals diagnosed with type 2 diabetes may fulfill the diagnostic criteria for an eating disorder, with Binge Eating Disorder (BED) being the predominant subtype [77].

Treatment for BED typically involves psychotherapy, such as cognitive-behavioral therapy or interpersonal therapy, and medication may also be used in some cases. In addition to these treatments, lifestyle changes such as improving sleep habits, increasing physical activity, and adopting healthy eating habits can also be helpful [78].

#### 2.3.2. Diarexia

Diarexia, sometimes referred to as “diabetic anorexia,” is another eating disorder commonly observed in individuals with diabetes, both type 1 and type 2. It involves the restrictive eating of food in an effort to maintain blood sugar control. People with diarexia may limit their food intake excessively, leading to inadequate nutrition and energy for proper diabetes management. They may prioritize blood sugar control over overall health and well-being, resulting in weight loss, malnutrition, and potentially severe complications. The patients usually intentionally reduce insulin intake, which leads to poor metabolic control and serious complications, such as neuropathy, retinopathy, nephropathy, and an increased incidence of hospital admissions due to ketoacidosis [79]. Comorbid anorexia with diabetes mellitus type 1 comes with a significantly raised mortality rate (34.7 per 1000) compared to patients with only anorexia (7.7 per 1000) or type 1 diabetes (2.2 per 1000) researched by Søren et al., 2002 [80]. Diarexia is a much lower incidence in diabetic females than diabulimia [42]; it is still a severe condition leading to an increased risk of complications and death.

#### 2.3.3. Night Eating Syndrome

Night Eating Syndrome (NES) is an eating disorder characterized by recurrent episodes of eating at night, either by waking up from sleep and eating or consuming a large amount of food after an evening meal. The clinical definition of NES, as described by Stunkard et al., 1955 [81], includes a triad of symptoms: morning anorexia (reduced appetite in the morning), evening hyperphagia (consuming 25% of total energy intake after 7 pm), and insomnia. If left untreated, NES can lead to difficulties in maintaining a healthy weight and may result in conditions such as type 2 diabetes and high blood pressure. The prevalence of NES is estimated to be around 1 in 100 individuals.

Research indicates that NES is more prevalent among individuals with type 2 diabetes, with prevalence rates ranging from 3.8% to 8.4%. Surprisingly, there may not be a significant difference in body mass index (BMI) between people with and without diagnosed NES, despite the association between NES and weight-related issues. NES may contribute to weight gain, particularly in individuals susceptible to obesity [82].

The exact causes of NES are not fully understood, but researchers believe it may result from various factors, including circadian rhythm disorders (disturbances in the body’s internal clock), depression and anxiety, daytime dieting, and genetic predisposition [83].

Treatment for NES typically involves a multidisciplinary approach, including psychotherapy and nutritional counseling. Behavioral therapies aim at establishing regular eating patterns, and addressing nighttime eating behaviors can be beneficial in managing NES. Additionally, identifying and addressing any underlying emotional or psychological factors is essential in the treatment process.

#### 2.3.4. Orthorexia Nervosa

Orthorexia nervosa (ON) is one of the unclassifiable eating behavior disorders and is briefly defined as an obsession with healthy eating [84]; it is an undeniably fast-spreading problem. Although not specific to diabetes, orthorexia is characterized by an exaggerated and prolonged obsession with adhering to strict dietary practices, often based on evidence-based or non-evidence-based information, in the pursuit of optimal health [85]. The distinguishing factor between healthy eating and ON lies in the severity of restrictive dietary behaviors and the level of self-discipline surrounding one’s diet. In ON, the focus is not solely on the quantity of food consumed but rather on the perceived quality of the food [86]; fixation can lead to obsessive behaviors related to sourcing, preparing, and consuming specific types of food, which may ultimately negatively impact various aspects of an individual’s life.

An exaggerated food intake restriction and the elimination of specific food groups, such as meat, dairy, grain, cooked food, and non-seasonal produce [87,88], may lead to severe complications such as a lack of essential nutrients and malnutrition, which can transform into gastrointestinal problems, hormonal imbalances, general fatigue, and a weakened immune system [89] but also anemia, or an abnormally slow heart rate [90].

Factors affecting the risk of ON and diabetes self-management can indeed be intertwined. People with Type 2 Diabetes Mellitus (T2DM) often have to follow strict dietary guidelines to effectively manage their blood glucose levels. This can include monitoring their carbohydrate intake, portion control, and adhering to specific meal plans. However, the focus on maintaining a healthy diet in diabetes management can sometimes lead to orthorexic tendencies. The strict rules and restrictions associated with diabetes self-management may trigger obsessive thoughts and behaviors around food quality and purity [91]. These individuals may become overly fixated on only consuming “healthy” foods and avoiding any perceived “unhealthy” or “impure” choices, which can contribute to the development or exacerbation of ON. Therefore, it is important to address and control orthorexic tendencies while simultaneously promoting effective diabetes self-management. This can be achieved through a balanced approach that focuses on education, support, and psychological well-being.

#### 2.3.5. Pregorexia

Pregorexia is a term that is informally used to describe disordered eating behaviors occurring during pregnancy. It is not officially recognized as a medical diagnosis in the Diagnostic and Statistical Manual of Mental Disorders (DSM) or other medical diagnostic manuals [92]. Some experts use the term pregorexia to refer to a subset of eating disorders, such as anorexia nervosa, bulimia nervosa, or binge eating disorder, that can either develop or worsen during pregnancy. However, there is an ongoing debate and lack of consensus among experts regarding the precise definition and diagnostic criteria for pregorexia. As a result, the term is not a standardized medical diagnosis. Women with preexisting eating disorders, such as anorexia, bulimia, or binge eating disorder, may experience challenges in managing their conditions during pregnancy. Pregnancy can trigger or exacerbate disordered eating behaviors, which can pose significant risks to both the mother and the developing fetus. For pregnant women with diabetes, there is also a potential risk of developing disordered eating behaviors. These behaviors can manifest as restrictive eating, extreme dieting, excessive exercise, or an unhealthy preoccupation with weight and body image during pregnancy. Disordered eating behaviors during pregnancy can lead to several adverse outcomes for both the mother and the baby. Nutritional deficiencies, such as iron deficiency leading to anemia, can affect the mother’s health and increase the risk of postpartum depression [93]. Additionally, these behaviors can result in increased risks of caesarian sections, miscarriage, low birth weight, microcephaly, and even infant mortality [94,95,96].

## 3. Treating Eating Disorders and Diabetes—The Dual Challenge

Treating individuals with both eating disorders and diabetes presents a dual challenge that necessitates a comprehensive approach that includes psychotherapy, glycemic control, nutritional counseling, and diabetes education [28]. For individuals with both type 2 diabetes and binge eating disorder (BED), daily challenges arise from conflicting medical and nutritional recommendations. While weight loss and energy deficit are emphasized to improve diabetes outcomes, BED treatment focuses on establishing a healthy relationship with food and reducing binge eating episodes [97]. Balancing these conflicting recommendations becomes difficult for those navigating both conditions. Hence, treatment teams must adopt an integrated approach that acknowledges and addresses both conditions, devising personalized strategies that cater to each individual’s unique needs. This approach takes into account the potential impact of dietary recommendations on both disorders, finding a middle ground between managing blood sugar levels and promoting a healthy relationship with food, ultimately enhancing overall well-being and quality of life. One of the considered methods is lifestyle behavioral changes, diet, behavioral therapy, and physical activity in order to obtain ≥5% weight loss and maintain it, which is recommended for patients with type 2 diabetes and obesity. It should include a high frequency of counseling and a more individual approach individualized to the patient. It needs to be focused on dietary changes, physical activity, and behavioral strategies to reach an energy deficit (500–750 kcal per day). For those who achieved weight loss goals, it is recommended to receive long-term maintenance programs [98]. Weight loss might not be enough to maintain it long-term. That people could benefit from additional pharmacotherapeutic options, such as Metformin, α-glucosidase inhibitors, liraglutide, thiazolidinediones, testosterone [99], and insulin [97]. Additionally, providers should consider the other drugs that patients use and, if possible, reduce or give alternatives for medications associated with weight gain, such as some antipsychotics, antidepressants etc. [100]. Prioritizing blood sugar level management is essential in diabetes care, and the treatment plan should include strategies for monitoring blood glucose levels, adjusting insulin dosages or types of antidiabetic medications, and ensuring adequate nutrition to maintain stable blood sugar levels. Dietitians play a critical role in providing education on balanced nutrition for managing diabetes while also addressing disordered eating patterns. Collaborating closely with individuals, dietitians develop personalized meal plans that meet nutritional needs and support the recovery process. It is important that recommendations should not be overly strict to avoid increasing the risk and frequency of binge eating episodes [21]. In cases where patients are overweight or obese, it is essential to prioritize treating the eating disorder before focusing on weight loss [101].

In individuals with type 1 diabetes, the presence of eating disorders often involves dangerous behaviors such as insulin restriction to control weight. However, this practice significantly increases the risk of complications. Therefore, treatment approaches should prioritize promoting healthy eating habits and regular blood glucose monitoring to manage diabetes effectively. Some individuals with eating disorders and type 1 diabetes may avoid testing their blood glucose regularly or altogether due to anxiety about high blood sugar levels. This behavior, known as test denial, can be an expression of anger or denial towards their illness. In severe cases, hospital admission may be necessary to control insulin use and stabilize eating habits. During hospitalization, nursing staff may temporarily take over insulin administration if concerns arise regarding the patient’s honesty about insulin administration.

What is more, a thorough assessment of the individual’s medical and psychiatric history, eating behaviors, blood sugar control, nutritional status, and mental health is essential. The psychological and emotional aspects of eating disorders can present challenges to the management of diabetes. Mental health conditions such as anxiety, depression, and distorted body image can influence self-care behaviors and adherence to diabetes treatment plans. In addressing these challenges, psychiatrists and psychopharmaceuticals, such as fluoxetine and topiramate, may be valuable in identifying and treating anxiety and binge eating episodes related to eating disorders. Emotional distress can also contribute to disordered eating patterns and unhealthy coping mechanisms. Several studies have reported that the fear of weight gain is a core component in the emergence of insulin mismanagement in T1DM patients [102]. Taking these factors into account is crucial for comprehensive care and successful management of both eating disorders and diabetes. Cognitive-behavioral therapy (CBT), dialectical behavior therapy (DBT), or interpersonal therapy are essential components of the treatment plan, helping individuals foster healthier relationships with food and their bodies. Cognitive behavioral therapy (CBT) has shown long-term effectiveness in reducing the frequency of binges and improving glycemic control, as demonstrated in studies by Kernardy et al., 2002 [103]. Therefore, it is crucial to address both conditions simultaneously for optimal outcomes. Regular follow-up visits and monitoring of progress are necessary to track the individual’s physical and mental health, make adjustments to the treatment plan as needed, and provide ongoing support [104].

Unfortunately, there is limited research on the psychological treatment of eating disorders in individuals with type 1 diabetes. Some studies have shown potential improvements when patients receive psychoeducation, family therapy, or cognitive-behavioral therapy (CBT). However, a recent systematic review suggested that these interventions might be ineffective in improving glycemic control [105]. Conventional CBT may yield inferior outcomes and higher dropout rates in individuals with both diabetes and bulimia compared to those with only bulimia nervosa [26]. Addressing the complex interplay between type 1 diabetes and eating disorders requires further research and the development of tailored interventions that consider the unique challenges posed by these dual conditions. Keep in mind that the presence of eating disorders in individuals with diabetes may be associated with an increased risk of mortality. A study conducted using female patient registries in Scandinavia investigated the mortality rates over a 10-year follow-up period. The findings revealed that among patients with type 1 diabetes, the mortality rate was 2.2 per 1000 person-years. In individuals with only anorexia, the mortality rate was 7.3 per 1000 person-years. Alarmingly, for patients who had both type 1 diabetes and anorexia concurrently, the mortality rate significantly increased to 34.6 per 1000 person-years [106]. These results emphasize the seriousness of the comorbidity and the importance of addressing both diabetes and eating disorders to ensure the well-being and longevity of affected individuals.

In conclusion, this review underscores the intricate connection between eating disorders and diabetes, shedding light on the imperative need to recognize and understand their multifaceted interplay. Eating disorders have become an unsettling and pervasive concern of health. The surge in their prevalence is influenced by a myriad of factors, including the rapid advancement of technology, the perpetuation of societal ideals propagated through diverse media platforms, low self-esteem, the prevalence of extreme dieting culture, genetic predisposition, and the presence of metabolic disorders such as diabetes. The symbiotic relationship between eating disorders and diabetes is characterized by a dynamic feedback loop wherein the presence of one condition can profoundly influence the course and outcomes of the other, resulting in a complex web of physiological, psychological, and behavioral interactions. Effectively addressing this complex issue demands early recognition and precisely targeted therapeutic intervention.

The insights gleaned from this comprehensive review may serve as a valuable wellspring of information for the development of new strategies that take into account the intricate dynamics shared by eating disorders and diabetes. The reflections gathered in this comprehensive review can provide a significant source of information for developing new strategies that address the complex dynamics common to eating disorders and diabetes. This review clearly highlights the importance of early diagnosis, shedding light on its potential benefits. As a result, healthcare professionals can increase their vigilance in detecting eating disorders and diabetes at an early stage. This is expected to translate into faster interventions that are often more effective and less invasive.

It is worth noting, however, that in addition to the diagnosis itself, the article also addresses the need for personalized therapeutic interventions in patients with eating disorders and diabetes. This approach may result in better treatment outcomes and increased adherence to treatment plans. The review also highlights the interdependence of these disorders, encouraging close collaboration between different healthcare professionals, such as nutritionists, endocrinologists, and mental health professionals. Working together, they can develop holistic treatment plans that take into account both the nutritional and psychological aspects of these conditions.

However, while this analysis takes into account the complexity of the problem, it does not provide specific recommendations or practical guidance for healthcare professionals. We realize that the article does not identify the gaps in previous research, which could be a starting point for future research and development in this field.

We realize that the article does not identify the gaps in previous research, which could be a starting point for future research and development in this field.

However, in our humble estimation, the publication of this review will have a significant impact on clinical practice by promoting early diagnosis, encouraging physicians to monitor patients for eating disorders and diabetes, and fostering interdisciplinary collaboration. The implementation of these changes in health care can have a significant impact on the quality of life of patients.

## Figures and Tables

**Table 1:** Signs of Disordered Eating in Diabetes.

Signs of Disordered Eating in Diabetes
Preoccupation with Food —Constant thoughts about food, calories, and weight
Strict Meal Plans —Following excessively rigid eating patterns and meal plans
Fear of High Blood Sugar —Extreme anxiety or fear of elevated blood sugar levels and their consequences
Distorted Body Image —Negative perception of body shape or size, even when it may not align with reality
Unhealthy Weight Control Behaviors —Engaging in extreme weight control methods, such as skipping meals or purging
Withdrawing from social eating activities —Knowingly or intentionally removing oneself from social gatherings or events that involve eating
Insulin Manipulation —Intentional omission or underdosing of insulin to manipulate weight or blood sugar levels
Excessive Exercise —Compulsive or excessive exercise routines as a means of compensating for food intake or controlling weight
Mood Swings or Emotional Distress —Frequent mood changes, anxiety, depression, or irritability related to food, body image, or diabetes management

**Table 2:** Types of Eating Disorders in Individuals with Diabetes.

Types of Eating Disorders in Individuals with Diabetes
1. Severe Dietary Restriction —Severe limitation of calories or essential macronutrients in the diet
2. Objective Binge Eating —Consuming an unusually large amount of food in a short period while feeling out of control
3. Subjective Binge Eating —Feeling a loss of control over eating without necessarily consuming large quantities of food
4. Other Maladaptive Weight Control Strategies —Restricting life-saving insulin

**Table 3:** Eating Disorders: Characteristics.

Eating Disorders
Anorexia Nervosa (AN) An unwillingness to maintain body weight above the minimum normal weight, which is typically around 85% of the expected weight or BMI is equal to or less than 17.5 for an individual’s height and age according to the ICD-10 classification. Severe disturbances in their body image, including a persistent fear of gaining weight or becoming fat, even when they are already underweight. Multiple physical complications, starting from the skin—xerosis, acne, telogen effluvium, through gastric dilatation with evidence of mucosal necrosis, arrhythmias, acrocyanosis, sodium depletion, and hypovolemia.
Avoidant/Restrictive Food Intake Disorder (ARFID) Limited interest or range of food choices, often due to sensory issues or negative experiences, Symptoms are lack of interest in food, avoidance based on sensory characteristics of food, and concern about aversive consequences of eating as examples of features.
Bulimia Nervosa (BN) Characterized by a cycle of binge eating followed by purging behaviors, Binge eating occurs at least twice a week for three months (DSM-5), Purging behaviors may include self-induced vomiting, fecal tenesmus, laxative use, or excessive exercise, Individuals may have a normal or slightly above-average body weight.
Binge-Eating Disorder (BED) Recurrent episodes of binge eating, during which a person eats a large amount of food in a short period of time and feels a lack of control over their eating, People with binge eating disorders do not engage in purging behaviors, strongly associated with overweight and obesity.
Diabulimia The intentional manipulation of insulin doses for the purpose of weight loss or weight control by individuals with type 1 diabetes, Severe hyperglycemia and related complications

**Table 4:** Diabulimia symptoms depend on its duration [66].

Diagnosed Duration	Symptoms
Short term	- polyuria with ketonuria, - polyphagia, - polydipsia, - high blood glucose (more than 250 mmol/L, but less than 600 mmol/L), - weakness, - fatigue - poor concentration, - unbalanced concentration of electrolytes (high potassium or low sodium)
Medium-term	- dehydration (moderate to severe), - high blood glucose (more than 250 mmol/L, but less than 600 mmol/L), - weight loss, - muscle atrophy, - indigestion, - gastroesophageal reflux disease, - edema
Long term	- high blood glucose (more than 250 mmol/L, but less than 600 mmol/L), - kidney damage (nephropathy), - neuropathy, - blindness (retinopathy), - extreme fatigue, - high levels of cholesterol conducing to cardiovascular problems, - osteoporosis

## Data Availability

Not applicable.

## References

[1] Weiselberg E.C., Gonzalez M., Fisher M. (2011). Eating disorders in the twenty-first century. Minerva Ginecol..

[2] Arcelus J., Mitchell A.J., Wales J., Nielsen S. (2011). Mortality rates in patients with anorexia nervosa and other eating disorders: A meta-analysis of 36 studies. Arch. Gen. Psychiatry.

[3] Santomauro D.F., Melen S., Mitchison D., Vos T., Whiteford H., Ferrari A.J. (2021). The hidden burden of eating disorders: An extension of estimates from the Global Burden of Disease Study 2019. Lancet Psychiatry.

[4] Hudson J.I., Hiripi E., Pope H.G., Kessler R.C. (2007). The prevalence and correlates of eating disorders in the National Comorbidiy Survey Replication. Biol. Psychiatry.

[5] Galmiche M., Déchelotte P., Lambert G., Tavolacci M.P. (2019). Prevalence of eating disorders over the 2000–2018 period: A systematic literature review. Am. J. Clin. Nutr..

[6] Robinson E., Higgs E. (2012). Liking food less: The impact of social influence on food liking evaluations in female students. PLoS ONE.

[7] Medical News Today (2016). Why Are Women More Vulnerable to Eating Disorders?. Brain Study Sheds Light..

[8] Engel S.G., Crosby R.D., Thomas G., Bond D., Lavender J.M., Mason T., Steffen K.J., Green D.D., Wonderlich S.A. (2016). Ecological momentary assessment in eating disorder and obesity research: A review of the recent literature. Curr. Psychiatry Rep..

[9] Crow S.J., Peterson C.B., Swanson S.A., Raymond N.C., Specker S., Eckert E.D., Mitchell J.E. (2019). Increased mortality in bulimia nervosa and other eating disorders. Am. J. Psychiatry.

[10] Garber A.K., Cheng J., Accurso E.C., Adams S.H., Buckelew S.M., Kapphahn C.J., Kreiter A., Le Grange D., Machen V.I., Moscicki A.-B. (2019). Weight loss and illness severity in adolescents with atypical anorexia nervosa. Pediatrics.

[11] Pisetsky E.M., Thornton L.M., Lichtenstein P., Pedersen N.L., Bulik C.M. (2013). Suicide attempts in women with eating disorders. J. Abnorm. Psychol..

[12] Halmi A.K. (2009). Anorexia nervosa: An increasing problem in children and adolescents. Dialogues Clin. Neurosci..

[13] Nicholls D.E., Lynn R., Viner R.M. (2011). Childhood eating disorders: British national surveillance study. Br. J. Psychiatry.

[14] Wojtyła A., Biliński P., Bojar I., Wojtyła C. (2011). Zaburzenia odżywiania u polskich gimnazjalistów. Prob. Hig. Epidemiol..

[15] Kołobo H., Woynarowska B. (2004). Samoocena masy ciała i odchudzanie się młodzieży w okresie dojrzewania. Prz. Pediatryczny.

[16] Hoek H.W. (2006). Incidence, prevalence and mortality of anorexia nervosa and other eating disorders. Curr. Opin. Psychiatry.

[17] Ogden C.L., Carroll M.D., Curtin L.R., McDowell M.A., Tabak C.J., Flegal K.M. (2006). Prevalence of overweight and obesity in the United States, 1999–2004. JAMA.

[18] Rabe-Jabłońska J., Pawełczyk T., Żechowski C., Jarema M. (2008). Standardy leczenia zaburzeń odżywiania. Therapeutic standards in eating disorders. Psychiatr. I Psychol. Klin..

[19] Smink R.R.E., Van Hoeken D., Hoek H.W. (2012). Epidemiology of eating disorders: Incidence, prevalence and mortality rates. Curr. Psychiatry Rep..

[20] Gabbri-Goebel A., Copeland P., Touyz S., Hay P. (2019). Editorial: Eating disorders in diabetes: Discussion on issues relevant to type 1 diabetes and an overview of the journal’s special issue. J. Eat. Disord..

[21] Gagnon C., Aimé A., Bélanger C., Markowitz J.T. (2012). Comorbid diabetes and eating disorders in adult patients. Diabetes Educ..

[22] Goebel-Fabbri A.E. (2008). Diabetes and eating disorders. J. Diabetes Sci. Technol..

[23] Rydall A.C., Rodin G.M., Olmsted M.P., Devenyi R.G., Daneman D. (1997). Disordered eating behavior and microvascular complications in young women with insulin-dependent diabetes mellitus. N. Engl. J. Med..

[24] Steel J.M., Young R.J., Lloyd G.G., Clarke B.F. (1987). Clinically apparent eating disorders in young diabetic women: Associations with painful neuropathy and other complications. Br. Med. J..

[25] Affenito S., Lammi-Keefe C., Vogel S., Backstrand J., Welch G., Adams C. (1997). Women with insulin-dependent diabetes mellitus (IDDM) complicated by eating disorders are at risk for exacerbated alterations in lipid metabolism. Eur. J. Clin. Nutr..

[26] Custal N., Arcelus J., Agüera Z., I Bove F., Wales J., Granero R., Jiménez-Murcia S., Sánchez I., Riesco N., Alonso P. (2014). Treatment outcome of patients with comorbid type 1 diabetes and eating disorders. BMC Psychiatry.

[27] Philpot U. (2013). Eating disorders in young people with diabetes: Development, diagnosis and management. J. Diabetes Nurs..

[28] Winston A.P. (2020). Eating Disorders and Diabetes. Curr. Diabetes Rep..

[29] Hanlan M.E., Griffith J., Patel N., Jaser S.S. (2013). Eating Disorders and Disordered Eating in Type 1 Diabetes: Prevalence, Screening, and Treatment Options. Curr. Diabetes Rep..

[30] Toni G., Berioli M.G., Cerquiglini L., Ceccarini G., Grohmann U., Principi N., Esposito S. (2017). Eating Disorders and Disordered Eating Symptoms in Adolescents with Type 1 Diabetes. Nutrients.

[31] Jones J.M., Lawson M.L., Daneman D., Olmsted M.P., Rodin G. (2000). Eating disorders in adolescent females with and without type 1 diabetes: Cross sectional study. BMJ.

[32] Grylli V., Hafferl-Gattermayer A., Schober E., Karwautz A. (2004). Prevalence and clinical manifestations of eating disorders in Austrian adolescents with type-1 diabetes. Wien Klin. Wochenschr..

[33] Young V., Eiser C., Johnson B., Brierley S., Epton T., Elliott J., Heller S. (2013). Eating problems in adolescents with Type 1 diabetes: A systematic review with meta-analysis. Diabet. Med..

[34] Meltzer L.J., Banks R.A., Bennett Johnson S., Prine J.M., Desrosiers P.M., Silverstein J.H. (2001). Disordered eating, body mass and glycemic control in adolescents with type 1 diabetes. Diabetes Care.

[35] Cass K., McGuire C., Bjork I., Sobotka N., Walsh K., Mehler P.S. (2020). Medical Complications of Anorexia Nervosa. Psychosomatics.

[36] Lilenfeld L.R., Kaye W.H., Greeno C.G., Merikangas K.R., Plotnicov K., Pollice C., Rao R., Strober M., Bulik C.M., Nagy L. (1998). A controlled family study of anorexia nervosa and bulimia nervosa: Psychiatric disorders in first-degree relatives and effects of proband comorbidity. Arch. Gen. Psychiatry.

[37] Strober M., Freeman R., Lampert C., Diamond J., Kaye W. (2000). Controlled family study of anorexia and bulimia nervosa: Evidence of shared liability and transmission of partial syndromes. Am. J. Psychiatry.

[38] Strober M., Freeman R., Lampert C., Diamond J., Kaye W. (2001). Males with anorexia nervosa: A controlled study of eating disorders in first-degree relatives. Int. J. Eat. Disord..

[39] Mitchell J.E., Crow S. (2006). Medical complications of anorexia nervosa and bulimia nervosa. Curr. Opin. Psychiatry.

[40] Martínez-González L., Fernández-Villa T., Molina A.J., Delgado-Rodríguez M., Martín V. (2020). Incidence of anorexia nervosa in women: A systematic review and meta-analysis. Int. J. Environ. Res. Public Health.

[41] Reas D.L., Ro O. (2018). Time trends in healthcare-detected incidence of anorexia nervosa and bulimia nervosa in the Norwegian national patient register. Int. J. Eat. Disord..

[42] Mannucci E., Rotella F., Ricca V., Moretti S., Placidi G.F., Rotella C.M. (2005). Eating disorders in patients with type 1 diabetes: A meta-analysis. J. Endocrinol. Investig..

[43] Bourne L., Bryant-Waugh R., Cook J., Mandy W. (2020). Avoidant/restrictive food intake disorder: A systematic scoping review of the current literature. Psychiatry Res..

[44] Ornstein R.M., Essayli J.H., Nicely T.A., Masciulli E., Lane-Loney S. (2017). Treatment of avoidant/restrictive food intake disorder in a cohort of young patients in a partial hospitalization program for eating disorders. Int. J. Eat. Disord..

[45] Fisher M.M., Rosen D.S., Ornstein R.M., Mammel K.A., Katzman D.K., Rome E.S., Callahan S.T., Malizio J., Kearney S., Walsh B.T. (2014). Characteristics of Avoidant/Restrictive Food Intake Disorder in Children and Adolescents: A “New Disorder” in DSM-5. J. Adolesc. Health.

[46] Forman S.F., McKenzie N., Hehn R., Monge M.C., Kapphahn C.J., Mammel K.A., Callahan S.T., Sigel E.J., Bravender T., Romano M. (2014). Predictors of outcome at 1 year in adolescents with DSM-5 restrictive eating disorders: Report of the national eating disorders quality improvement collaborative. J. Adolesc. Health.

[47] Nicely T.A., Lane-Loney S., Masciulli E., Hollenbeak C.S., Ornstein R. (2014). Prevalence and characteristics of avoidant/restrictive food intake disorder in a cohort of young patients in day treatment for eating disorders. J. Eat. Disord..

[48] Norris M.L., Robinson A., Obeid N., Harrison M., Spettigue W., Henderson K. (2014). Exploring avoidant/restrictive food intake disorder in eating disordered patients: A descriptive study. Int. J. Eat. Disord..

[49] Williams K.E., Hendy H.M., Field D.G., Belousov Y., Riegel K., Harclerode W. (2015). Implications of avoidant/restrictive food intake disorder (ARFID) on children with feeding problems. Child. Health Care.

[50] Cooney M., Lieberman M., Guimond T., Katzman D.K. (2018). Clinical and psychological features of children and adolescents diagnosed with avoidant/restrictive food intake disorder in a pediatric tertiary care eating disorder program: A descriptive study. J. Eat. Disord..

[51] Krom H., van der Sluijs Veer L., van Zundert S., Otten M., Benninga M., Haverman L., Kindermann A. (2019). Health related quality of life of infants and children with avoidant restrictive food intake disorder. Int. J. Eat. Disord..

[52] Hay P., Mitchison D., Collado A.E.L., Gonzalez-Chica D.A., Stocks N., Touyz S. (2017). Burden and health-related quality of life of eating disorders, including avoidant/restrictive food intake disorder (ARFID), in the Australian population. J. Eat. Disord..

[53] Gonçalves S., Vieira A.I., Machado B.C., Costa R., Pinheiro J., Conceiçao E. (2018). Avoidant/restrictive food intake disorder symptoms in children: Associations with child and family variables. Child. Health Care.

[54] Chen Y., Chen W.J., Lin K., Shen L., Gau S.S. (2019). Prevalence of DSM-5 mental disorders in a nationally representative sample of children in Taiwan: Methodology and main findings. Epidemiol. Psychiatr. Sci..

[55] Norris M.L., Spettigue W.J., Katzman D.K. (2016). Update on eating disorders: Current perspectives on avoidant/restrictive food intake disorder in children and youth. Neuropsychiatr. Dis. Treat..

[56] Rzońca E., Bień A., Iwanowicz-Palus G. (2016). Zaburzenia odżywiania-problem wciąż aktualny. J. Educ. Health Sport.

[57] Osińska A., Mozol-Jursza M., Tyszkiewicz-Nwafor M., Słopień A., Paszyńska E. (2016). Bulimia psychiczna–rozpowszechnienie, objawy i leczenie z uwzględnieniem aspektu stomatologicznego. Pediatr. I Med. Rodz..

[58] Blake K., Davis V., Marcdante K.J., Kliegman R.M., Jenson H.B., Behrman R.E. (2013). Medycyna okresu dorastania. Nelson Pediatria.

[59] Zerwas S., Larsen J.T., Petersen L., Thornton L.M., Mortensen P.B., Bulik C.M. (2015). The incidence of eating disorders in a Danish register study: Associations with suicide risk and mortality. J. Psychiatr. Res..

[60] Hoek H.W. (2021). Incidence, prevalence and mortality of anorexia nervosa and bulimia nervosa. Curr. Opin. Psychiatry.

[61] Himmerich H., Hotopf M., Shetty H., Schmidt U., Treasure J., Hayes R.D., Stewart R., Chang C.-K. (2019). Psychiatric comorbidity as a risk factor for the mortality of people with bulimia nervosa. Soc. Psychiatry Psychiatr. Epidemiol..

[62] Forcano L., Fernández-Aranda F., Álvarez-Moya E., Bulik C., Granero R., Gratacòs M., Jiménez-Murcia S., Krug I., Mercader J.M., Riesco N. (2019). Suicide attempts in bulimia nervosa: Personality and psychopathological correlates. Eur. Psychiatry.

[63] Brown C.A., Mehler P.S. (2013). Medical complications of self-induced vomiting. Eat. Disord..

[64] Herpertz S., Albus C., Lohff S., Michalski K., Masrour M., Lichtblau K., Köhle K., Mann K., Senf W. (2000). Characteristics of diabetic patients with and without an eating disorder. Psychother. Psychosom. Med. Psychol..

[65] Vila G., Robert J.-J., Nollet-Clemencon C., Vera L., Crosnier H., Rault G., Jos J., Mouren-Simeoni M.-C. (1995). Eating and emotional disorders in adolescent obese girls with insulin-dependent diabetes mellitus. Eur. Child Adolesc. Psychiatry.

[66] Ruth-Sahd L.A., Schneider M., Haagen B. (2009). Diabulimia: What It Is and How to Recognize It in Critical Care. Dimens. Crit. Care Nurs..

[67] Colton P.A., Olmsted M.P., Daneman D., Farquhar J.C., Wong H., Muskat S., Rodin G.M. (2015). Eating disorders in girls and women with type 1 diabetes: A longitudinal study of prevalence, onset, remission, and recurrence. Diabetes Care.

[68] Mather A.A., Cox B.J., Enns M.W., Sareen J. (2009). Associations of obesity with psychiatric disorders and suicidal behaviors in a nationally representative sample. J. Psychosom. Res..

[69] Bădescu S., Tătaru C., Kobylinska L., Georgescu E., Zahiu D., Zăgrean A., Zăgrean L. (2016). The association between Diabetes mellitus and Depression. J. Med. Life.

[70] Kessler R.C., Berglund P.A., Chiu W.T., Deitz A.C., Hudson J.I., Shahly V., Aguilar-Gaxiola S., Alonso J., Angermeyer M.C., Benjet C. (2013). The Prevalence and Correlates of Binge Eating Disorder in the World Health Organization World Mental Health Surveys. Biol. Psychiatry.

[71] Giel K.E., Bulik C.M., Fernandez-Aranda F., Hay P., Keski-Rahkonen A., Schag K., Schmidt U., Zipfel S. (2022). Binge eating disorder. Nat. Rev. Dis. Primers.

[72] Hilbert A. (2019). Binge-eating disorder. Psychiatr. Clin..

[73] García-Mayor R.V., García-Soidán F.J. (2017). Eating disorders in type 2 diabetic people: Brief review. Diabetes Metab. Syndr..

[74] Papelbaum M., Appolinário J.C., Moreira Rde O., Ellinger V.C., Kupfer R., Coutinho W.F. (2005). Prevalence of eating disorders and psychiatric comorbidity in a clinical sample of type 2 diabetes mellitus patients. Braz. J. Psychiatry.

[75] Herpertz S., Albus C., Wagener R., Kocnar M., Wagner R., Henning A., Best F., Foerster H., Schleppinghoff B.S., Thomas W. (1998). Comorbidity of diabetes and eating disorders. Does diabetes control reflect disturbed eating behavior?. Diabetes Care.

[76] Abbott S., Dindol N., Tahrani A.A., Piya M.K. (2018). Binge eating disorder and night eating syndrome in adults with type 2 diabetes: A systematic review. J. Eat. Disord..

[77] Raevuori A., Suokas J., Haukka J., Gissler M., Linna M., Grainger M., Suvisaari J. (2015). Highly increased risk of type 2 diabetes in patients with binge eating disorder and bulimia nervosa. Int. J. Eat. Disord..

[78] Dingemans A.E., Bruna M.J., van Furth E.F. (2002). Binge eating disorder: A review. Int. J. Obes..

[79] Winston A.P. (2012). The clinical biochemistry of anorexia nervosa. Ann. Clin. Biochem..

[80] Nielsen S., Emborg C., Mølbak A.G. (2002). Mortality in Concurrent Type 1 Diabetes and Anorexia Nervosa. Diabetes Care.

[81] Stunkard A., Grace W., Wolff H. (1955). The night-eating syndrome: A pattern of food intake among certain obese patients. Am. J. Med..

[82] Gallant A.R., Lundgren J., Drapeau V. (2012). The night-eating syndrome and obesity. Obes. Rev..

[83] Night Eating Syndrome (NES). https://my.clevelandclinic.org/health/diseases/21731-night-eating-syndrome-nes.

[84] Varga M., Dukay-Szabó S., Túry F., van Furth Eric F. (2013). Evidence and gaps in the literature on orthorexia nervosa. Eat. Weight Disord. -Stud. Anorex. Bulim. Obes..

[85] Hanganu-Bresch C. (2020). Orthorexia: Eating right in the context of healthism. Med. Humanit..

[86] Uzdil Z., Kayacan A.G., Özyildirim C., Seda K., Kilinç G.E., Canan A., Kaya P.S. (2019). Adölesanlarda Ortoreksiya Nervoza Varlığı ve Yeme Tutumunun İncelenmesi. Samsun Sağlık Bilim. Derg..

[87] Bratman S., Knight D. (2004). Health Food Junkies: Orthorexia Nervosa: Overcoming the Obsession with Healthful Eating.

[88] Donini L.M., Marsili D., Graziani M.P., Imbriale M., Cannella C. (2004). Orthorexia nervosa: A preliminary study with a proposal for diagnosis and an attempt to measure the dimension of the phenomenon. Eat. Weight Disord. -Stud. Anorex. Bulim. Obes..

[89] Oberle C.D., Klare D.L., Patyk K.C. (2019). Health beliefs, behaviors, and symptoms associated with orthorexia nervosa. Eat. Weight Disord. Stud. Anorexia. Bulim. Obes..

[90] Koven N.S., Abry A.W. (2015). The clinical basis of orthorexia nervosa: Emerging perspectives. Neuropsychiatr. Dis. Treat..

[91] Altun H.K., Özyildirim C., Koç Ş., Aksoy H.N., Sağir B., Bozkurt M.S., Karasu H. (2023). The factors associated with orthorexia nervosa in type 2 diabetes and their effect on diabetes self-management scores. Eat. Weight Disord..

[92] Tunçere E., Bayindirgümüş A. (2020). The Importance of Pregorexia Awareness. Clin. Exp. Health Sci..

[93] Wójciak R.W., Mojs E., Michalska M.M., Samulak D. (2013). Podejmowanie odchudzania w okresie ciąży a poporodowe surowicze stężenia żelaza u kobiet–badanie wstępne. Probl. Hig. Epidemiol..

[94] Micali N. (2008). Eating disorders and pregnancy. Psychiatry.

[95] Bee H., Boyd D. (2004). The Developing Child.

[96] Eik-Nes T.T., Horn J., Strohmaier S., Holmen T.L., Micali N., Bjornelv S. (2018). Impact of eating disorders on obstetric outcomes in a large clinical sample: A comparison with the HUNT study. Int. J. Eat. Disord..

[97] American Diabetes Association Professional Practice Committee (2022). 3. Prevention or Delay of Type 2 Diabetes and Associated Comorbidities: Standards of Medical Care in Diabetes—2022. Diabetes Care.

[98] American Diabetes Association Professional Practice Committee (2022). 8. Obesity and Weight Management for the Prevention and Treatment of Type 2 Diabetes: Standards of Medical Care in Diabetes–2022. Diabetes Care.

[99] Wittert G., Bracken K., Robledo K.P., Grossmann M., Yeap B.B., Handelsman D.J., Stuckey B., Conway A., Inder W., McLachlan R. (2021). Testosterone treatment to prevent or revert type 2 diabetes in men enrolled in a lifestyle programme (T4DM): A randomised, double-blind, placebo-controlled, 2-year, phase 3b trial. Lancet Diabetes Endocrinol..

[100] Domecq J.P., Prutsky G., Leppin A., Sonbol M.B., Altayar O., Undavalli C., Wang Z., Elraiyah T., Brito J.P., Mauck K.F. (2015). Clinical review: Drugs commonly associated with weight change: A systematic review and meta-analysis. J. Clin. Endocrinol. Metab..

[101] Larrañaga A., Fluiters E., Docet M., Fernández Sastre J.L., García-Mayor R.V. (2014). Comparative study of cognitive-behavioural psychotherapy and nutritional support in patients with different types of eating disorders. Med. Clin..

[102] Coleman S.E., Caswell N. (2020). Diabetes and eating disorders: An exploration of ‘Diabulimia’. BMC Psychol..

[103] Kernardy J., Mensch M., Bowen K., Green B., Watson J. (2002). Group therapy for binge eating in type 2 diabetes: A randomized trial. Diabet. Med..

[104] Lehman R., Krumholz H.M. (2009). Tight control of blood glucose in long standing type 2 diabetes. BMJ.

[105] Clery P., Stahl D., Ismail K., Treasure J., Kan C. (2017). Systematic review and meta-analysis of the efficacy of interventions for people with Type 1 diabetes mellitus and disordered eating. Diabet. Med..

[106] Nielsen S. (2002). Eating disorders in females with type 1 diabetes: An update of a metaanalysis. Eur. Eat. Disord. Rev..

